# iCN718, an Updated and Improved Genome-Scale Metabolic Network Reconstruction of *Acinetobacter baumannii* AYE

**DOI:** 10.3389/fgene.2018.00121

**Published:** 2018-04-10

**Authors:** Charles J. Norsigian, Erol Kavvas, Yara Seif, Bernhard O. Palsson, Jonathan M. Monk

**Affiliations:** Department of Bioengineering, University of California, San Diego, San Diego, CA, United States

**Keywords:** *Acinetobacter baumannii*, genome-scale reconstruction, antibiotic resistance, constraint-based modeling, metabolism

## Abstract

*Acinetobacter baumannii* has become an urgent clinical threat due to the recent emergence of multi-drug resistant strains. There is thus a significant need to discover new therapeutic targets in this organism. One means for doing so is through the use of high-quality genome-scale reconstructions. Well-curated and accurate genome-scale models (GEMs) of *A. baumannii* would be useful for improving treatment options. We present an updated and improved genome-scale reconstruction of *A. baumannii* AYE, named iCN718, that improves and standardizes previous *A. baumannii* AYE reconstructions. iCN718 has 80% accuracy for predicting gene essentiality data and additionally can predict large-scale phenotypic data with as much as 89% accuracy, a new capability for an *A. baumannii* reconstruction. We further demonstrate that iCN718 can be used to analyze conserved metabolic functions in the A. baumannii core genome and to build strain-specific GEMs of 74 other *A. baumannii* strains from genome sequence alone. iCN718 will serve as a resource to integrate and synthesize new experimental data being generated for this urgent threat pathogen.

## Introduction

*Acinetobacter baumannii* has recently emerged as a deadly nosocomial threat with rising rates of both infection and antibiotic resistance. Reports using data from hospital-based surveillance studies as well as from the Infectious Diseases Society of America have begun to refer to a dangerous group of nosocomial pathogens, including *A. baumannii*, as “ESKAPE pathogens” (Rice, 2008). *A. baumannii* in particular is known for its highly persistent and opportunistic nature, most often resulting in hospital-acquired pneumonia while also having the ability to infect various other tissues (Weber et al., 2015). Organisms of the genus *Acinetobacter* inhabit a wide variety of environments, ranging from humans to water and soil (Vallenet et al., 2008). These diverse environmental niches are reflected in the genomic content of the organisms as well as their metabolic capabilities. *Acinetobacter* are Gram-negative, aerobic, and non-motile. Pathogenic *A. baumannii* antibiotic resistance has risen from a susceptible level in the 1960s to extended and pan-drug resistant today (Peleg et al., 2008). As such, the need for new treatment targets and strategies is dire.

Genome-scale models (GEMs) of metabolism have been used to discover new drug targets (Kim et al., 2011) and pursue novel treatment options. Genome-scale metabolic reconstructions offer an established framework for systems-level analyses of an organism’s metabolism (Thiele and Palsson, 2010). GEMs provide a formal way to link genotype to phenotype and mechanistically analyze the metabolic capabilities of organisms. A previous reconstruction of the metabolic network of *A. baumannii* AYE was undertaken and produced: AbyMBEL891 (Kim et al., 2010). This reconstruction provided a valuable starting point for the progress and use of GEMs to study the pathogenic nature of *A. baumannii*. However, one issue that has limited the use of this and other reconstructions is the lack of standardization in identifiers for metabolites and reactions (Ebrahim et al., 2015). Since the publication of AbyMBEL891 in 2010, numerous studies have produced new data (Farrugia et al., 2013; Gallagher et al., 2015; Presta et al., 2017) that provide an opportunity to update this *A. baumannii* reconstruction, allowing for more accurate representations of its physiology. One such study was a high-quality reconstruction of *A. baumannii* ATCC 19606, iLP844, that served as a valuable resource for model improvements (Presta et al., 2017). Furthermore, given that *Acinetobacter* is known to populate a diverse array of environments, particularly hospitals, it is likely that diverse metabolic capabilities may be present throughout the different strains in this species.

We present iCN718, a new and updated GEM of *A. baumannii* AYE (**Supplementary Datasheets S1–S3**). This reconstruction utilizes AbyMBEL891 as a foundation. We validated our model by comparing phenotypic predictions made by iCN718 to those made by AbyMBEL891. We extended our analysis to additional datasets published after AbyMBEL891. We assessed iCN718 on its ability to predict both gene essentiality and to recapitulate experimental growth capabilities. We then utilize this reconstruction to create draft models of 74 other *A. baumannii* strains from their sequence data alone. We leverage the reconstruction to produce draft models to gain insight into these other strains and the species as a whole. Thus, iCN718 offers a framework for sequence-to-model comparisons. Our updated model of *A. baumannii* will provide new opportunities to advance the understanding of pathogenic microbes and their interactions with human hosts.

## Results and Discussion

### Workflow for Network Reconstruction

**FIGURE 1:**
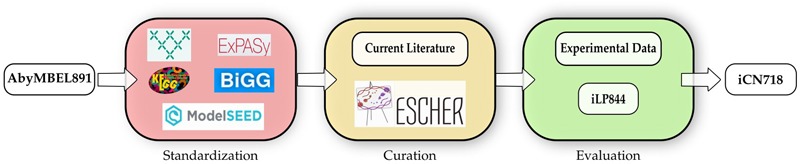
Workflow of the reconstruction process. The starting reconstruction, AbyMBEL891, was cross referenced against a draft model generated utilizing ModelSEED (Henry et al., 2010). Next, the reconstruction was standardized using various databases mapped to standard BIGGs IDs. This process was followed by manual curation based on current literature on the organism, aided by the use of ESCHER to visualize pathways throughout the process. Finally, the model was evaluated against experimental datasets and compared to iLP844 a model of *Acinetobacter baumannii* ATCC 19606 to further improve the reconstruction. The model was iteratively evaluated against gene essentiality and phenotypic datasets to improve the reconstruction accuracy.

We began the metabolic network reconstruction process by updating AbyMBEL891. We found that the AbyMBEL891 reconstruction could be updated and improved in three main areas: (1) standardization of reaction and metabolite identifiers to increase the tractability of the network, (2) mass and charge balance metabolic reactions, and (3) transport processes. Before updating and improving the reconstruction, we recognized that it was necessary to translate AbyMBEL891 into a format that could be more readily analyzed. We obtained a draft reconstruction of *A. baumannii* AYE using the ModelSeed database (Henry et al., 2010). We then cross-referenced draft reconstruction reactions against AbyMBEL891 and utilized additional databases to map all reactions and metabolites to the standardized BiGG format (King et al., 2016). Additionally, we added the curated gene product rules (GPRs) from AbyMBEL891 into iCN718 to improve ease of simulation (Presta et al., 2017). The resulting model was then continually and iteratively improved through manual curation of new organism knowledge in the literature published since the release of AbyMBEL891 (See section “Materials and Methods” and **Figure 1**).

iCN718 comprises 718 genes, 1016 reactions, and 890 metabolites compared to the 650 genes, 891 reactions, and 770 metabolites in AbyMBEL891. The majority of the difference in reactions included arises from the inclusion of exchange reactions in iCN718 as well as revamping the transport reactions. The reversibility of reactions within iCN718 was referenced against the reversibility of corresponding reactions in a recently published model of *A. baumannii* ATCC 19606, iLP844 (Presta et al., 2017). In some cases, reaction reversibility was changed to reflect the state in iLP844. Reversibility was corroborated with iLP844 for a set of about 50 reactions and edited accordingly. iLP844 was also used to identify GPRs for transport reactions present in both models, leading to the inclusion of 66 new genes in iCN718. Further, new reactions that were missing in the original reconstruction were added in peptidoglycan biosynthesis, propanoate metabolism, and glycolate catabolism. The end product of iCN718 is a reconstruction of *A. baumannii* AYE that rectifies issues with AbyMBEL891 regarding identifiers, reversibility of reactions, transport/exchange reactions, and mass/charge balancing. Well-curated identifiers were added for every reaction in the network. Thus, iCN718 provides an improved knowledge-base for the study of *A. baumannii*.

After completing the reconstruction of iCN718, we calculated the metabolite connectivity to evaluate the network structure for both iCN718 and AbyMBEL891 (Becker et al., 2006). Metabolite connectivity refers to the number of reactions in which a metabolite participates. Given that metabolites are the nodes of the network connected by reactions, this metric reveals the connectivity of a metabolic network. We compared the metabolite connectivities of iCN718 and AbyMBEL891 (**Supplementary Figure S1**) and found that overall, the networks were comparable, but these plots do not visualize dead-end metabolites (i.e., metabolites with a connectivity of one). iCN718 has four dead-end metabolites whereas AbyMBEL891 has 145 dead-end metabolites, demonstrating that iCN718 is more highly connected overall. The increase in connectivity is a result of converting to BiGG standard identifiers which improves the regularity of the network.

### Functional Evaluation of iCN718

**FIGURE 2:**
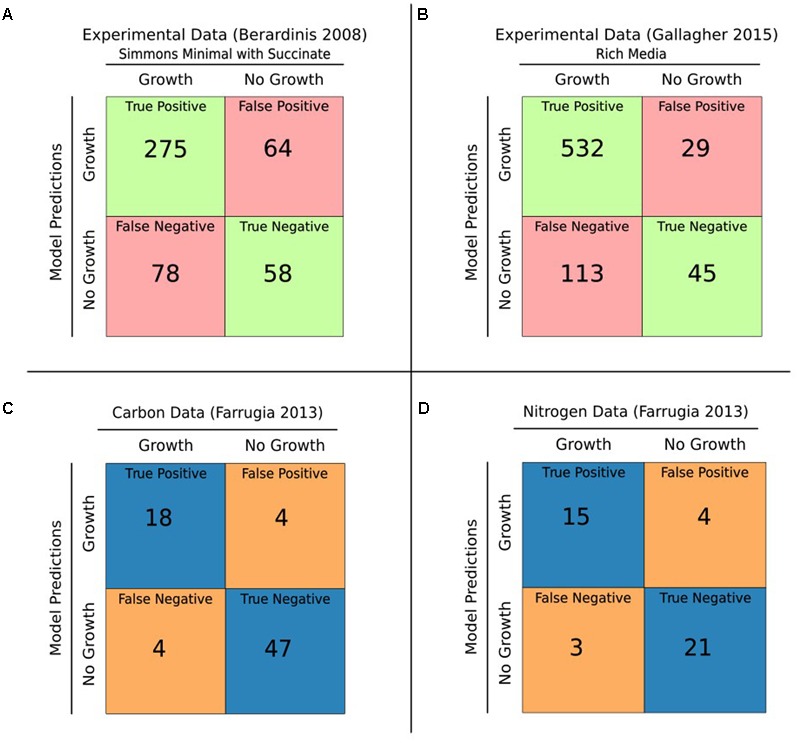
Gene essentiality and growth predictions. **(A)** iCN718 was used to predict gene essentiality. The results were compared to the de Berardinis et al. (2008) experimental dataset with 68% accuracy. **(B)** iCN718 predicted gene essentiality results compared with the Gallagher et al. (2015) dataset exhibited 80% accuracy. It is worth noting that the Berardinis dataset was of *Acinetobacter baylyi* ADP1 and therefore not every gene in iCN718 had an orthologous gene in the essentiality dataset. Green represents correct predictions, red represents incorrect predictions. The Gallagher dataset is from *Acinetobacter baumannii* strain AB5075 of which there is an ortholog for every gene within iCN718. Model-predicted ability to catabolize various sole carbon **(C)** and sole nitrogen **(D)** sources compared to the Farrugia et al. (2013) Biolog Phenotypic Array data for *Acinetobacter baumannii* AYE exhibited 89% and 84% accuracy, respectively. Blue represents correct predictions, orange represents incorrect predictions. Only compounds readily mapped to model metabolites were included from the Biolog data.

Our first functional evaluation of iCN718 consisted of analyzing its accuracy in predicting gene essentiality for three datasets (**Figures 2A,B**). The most comprehensive essentiality dataset available was used (Gallagher et al., 2015). This complete TN-seq essentiality dataset was conducted with *A. baumannii* AB5075 and is particularly valuable because it is of genome scale and every gene in iCN718 has an ortholog. iCN718 was able to achieve 80.22% accuracy (**Figure 2B**). Unfortunately, given the lack of GPRs in AbyMBEL891, we were unable to analyze its performance on this dataset. We also evaluated iCN718’s performance on the two datasets originally used to validate AbyMBEL891. The first was an insertional mutagenesis dataset with *A. baumannii* ATCC 19606 by Dorsey et al. (2002) on a set of 14 mutants. We repeated the same knockouts *in silico* as done in the original experiment and found that iCN718 was able to correctly predict 100% (14/14) of the mutant cases as did AbyMBEL891. The obvious limitation of this dataset is that it is on such a small scale. The second dataset used to validate AbyMBEL891, by de Berardinis et al. (2008), was a complete, genome-scale set of single-gene deletions in *Acinetobacter baylyi* ADP1. iCN718 fell short in predictive ability on this dataset compared to AbyMBEL891 (**Figure 2A**), with 68% and 72% accuracy, respectively.

The higher predictive accuracy on the Gallagher dataset compared to the de Beradinis dataset is encouraging because strain AB5075 is a clinical isolate like AYE whereas *A. baylyi* ADP1 is a soil strain. The disparity in genomic content between *A. baumannii* AYE and *A. baylyi* ADP1 is evident in the limited number of genes in iCN718 that have an ortholog. Despite the limitations of the original two datasets, whether it be scale or lack of similarity, it was important to test iCN718’s ability to recapitulate the capabilities of AbyMBEL891. Overall, iCN718 performed the same as AbyMBEL891 on the datasets originally used for validation. Further, there is more agreement of genes with a dataset on a strain that is closer to the target of the reconstruction. It is reasonable to conclude from these gene-essentiality results that at a minimum, iCN718 performs in line with AbyMBEL891 in regard to gene essentiality and more likely is superior in predictive capability. An obvious avenue for further improvement of the reconstruction would be to develop a gene essentiality dataset for strain AYE.

We further extended our assessment of iCN718 to large-scale phenotypic data. By utilizing the Biolog Phenotype Microarray data published by Farrugia et al. (2013), we were able to iteratively improve iCN718 through manual curation for discrepancies. The model had encouraging agreement at the end of this process for sole carbon and nitrogen sources readily tractable to the model (116 total; **Figures 2C,D**). Growth rates were calculated in Simmons’ Minimal Medium and iteratively investigated for each carbon or nitrogen source in the microarray wet lab experiment. The model result of growth or no growth determined by optimizing for the biomass function was compared to the data from the microarray (**Supplementary Tables S1, S2**). For the carbon sources tested on the microarray plate, 73 metabolites were analyzed and showed that iCN718 has 89.1% agreement with the experimental data. Likewise, for nitrogen sources, 43 metabolites were screened with 83.7% agreement. Importantly, out of all the datasets used for validation of the reconstruction, this microarray data was the only set executed with the strain of interest, *A. baumannii* AYE. Therefore, this dataset was particularly valuable for insight into the capabilities of this specific strain.

**FIGURE 3:**
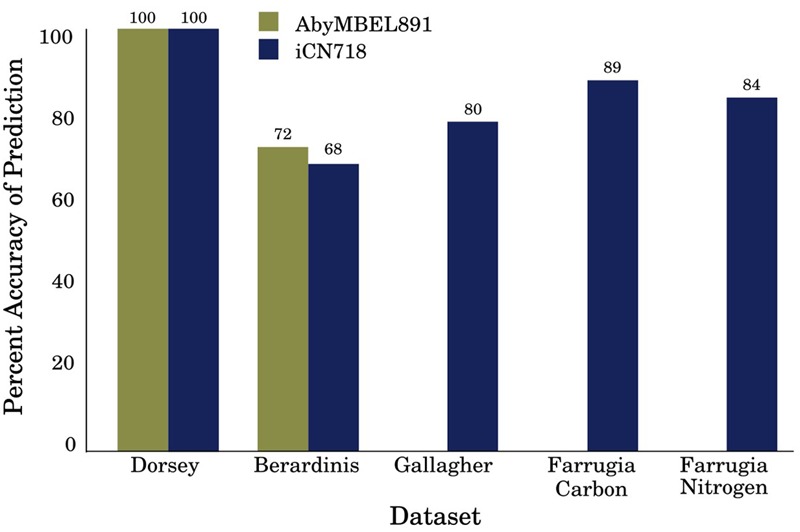
Summary of AbyMBEL891 and iCN718 Performance. Overall performance of iCN718 compared to a previous *Acinetobacter baumannii* AYE reconstruction (AbyMBEL891). Both models perform similarly on the datasets originally used to validate AbyMBEL891; however, the ability to simulate sole carbon and nitrogen sources in minimal media is exclusive to iCN718. AbyMBEL891 could not be simulated with the Gallagher dataset and was incapable of growth in the conditions of the Farrugia dataset.

We have demonstrated that iCN718 performs as well as AbyMBEL891 on datasets originally used to validate AbyMBEL891. We note that these datasets suffer from limitations in that they are either not genome scale or are not of an ideally similar species to the strain of interest. To expand the validation of iCN718 and address these limitations, we analyzed a genome-scale set of gene essentiality data of another *A. baumannii* clinical strain and found a reasonably high level of agreement. Further we analyzed iCN718’s agreement with phenotypic microarray experiments conducted with strain AYE. iCN718’s ability to capture this growth behavior is a major improvement over AbyMBEL891, which fails to simulate on the minimal media conditions corresponding to these experiments. Overall, we showed that iCN718 maintains comparable performance on the original datasets used for validation, has a higher agreement with gene essentiality data for a more closely related strain, and is able to correctly predict phenotypic growth experiments (**Figure 3**). We used the model to perform synthetic lethals analysis to generate new predictions. Briefly this resulted in 49 synthetic lethal gene pairs that include 62 unique genes. These genes correspond to reactions involved in fatty acid metabolism, purine metabolism, glycine/serine/threonine metabolism, phenylalanine/tyrosine/tryptophan biosynthesis, TCA cycle, lysine degradation, glycerophospholipid metabolism, glycolysis, pyrimidine metabolism, nicotinate/nicotinamide metabolism, riboflavin metabolism, pentose phosphate pathway, cysteine metabolism, and methionine metabolism. Full double-gene deletion results and synthetic lethal gene pairs are reported in **Supplementary Tables S4** and **S5**, respectively.

### Pan-Genome Analysis of *A. baumannii* Using iCN718

**FIGURE 4:**
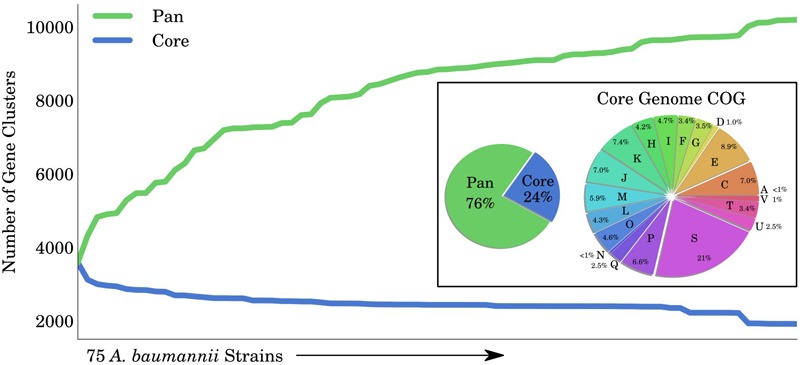
Pan and Core Genome of *Acinetobacter baumannii*. The total number of gene clusters in 75 *Acinetobacter baumannii* strains (pan-genome) compared to those that are shared among all strains (core-genome). In total, 76% of the clusters are classified as accessory and 24% as core. The core genome was functionally classified into COG categories. COG categories are as follows: Cellular processes and signaling: D is cell cycle control, cell division, and chromosome partitioning; M is cell wall/membrane/envelope biogenesis; N is cell motility; O is posttranslational modification, protein turnover, and chaperones; T is signal transduction mechanisms; U is intracellular trafficking, secretion, and vesicular transport; V is defense mechanisms; W is extracellular structures; Y is nuclear structure; and Z is cytoskeleton. Information storage and processing: A is RNA processing and modification; B is chromatin structure and dynamics; J is translation, ribosomal structure, and biogenesis; K is transcription; and L is replication, recombination, and repair. Metabolism: C is energy production and conversion; E is amino acid transport and metabolism; F is nucleotide transport and metabolism; G is carbohydrate transport and metabolism; H is coenzyme transport and metabolism; I is lipid transport and metabolism; P is inorganic ion transport and metabolism; and Q is secondary metabolite biosynthesis, transport, and catabolism.

A GEM can be used to investigate the capabilities of organisms across multiple strains. We applied these principles using iCN718 to explore the different genotypes and phenotypes within the *A. baumannii* species. There are 75 full complete sequences of *A. baumannii* available on the PATRIC database (Wattam et al., 2014); these range from a wide variety of isolation countries and are largely isolates from a clinical/human setting (See **Supplementary Table S6**). We collected the annotated open reading frames (ORFs) from each of these genomes and used CD-HIT (Fu et al., 2012) to assign their coding sequences into clusters of at least 80% similarity. Clusters that were found in at least 74 of the 75 strains were determined to be core genes, while those found in only some of the strains were designated as accessory genes. In total, 24% (2448/10200) of the genes were found across all strains (core genome) while 76% (7752/10200) were part of the accessory genome (**Figure 4**). We further classified the core genome by clusters of orthologous groups (COGs) and found that while a large group (21%) had unknown functions, the remaining 79% of the core genome had a widely varied classification spanning 19 other COG categories. Overall the core genome had ∼33% COGs pertaining to metabolic functions. Particularly interesting was that 8.9% of the core genome was composed of functions in amino acid transport and metabolism (category E), suggesting that this area of metabolism might be particularly conserved over these strains of *A. baumannii*. We also classified the pan genome and note that roughly half could not be COG classified and almost half of that classified portion was classified as having unknown function (**Supplementary Figure S2**). This suggests that more robust study and classification of these strains is necessary.

**FIGURE 5:**
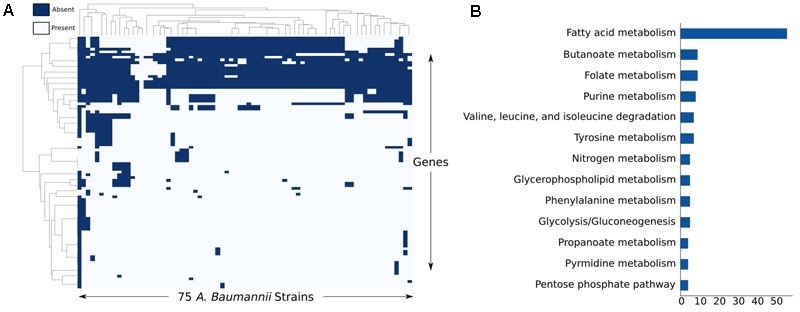
Analysis of least conserved genes. **(A)** Clustermap of the genes most deleted from each strain-specific model and **(B)** the corresponding subsystems of the reactions these genes code for.

After analyzing the full set of annotated ORFs across the 75 strains, we were particularly interested in applying the iCN718 reconstruction to construct draft strain-specific models of them. To accomplish building these draft models, we determined presence or absence of the 718 genes in the reconstruction of AYE and deleted genes accordingly for the other 74 strains (See **Supplementary Table S7**). After this process, we had a measure of the “metabolic pan-genome” as it relates to the genes contained within iCN718. Utilizing the same thresholds, we found that 86% of the genes in iCN718 were considered to be core to all 74 additional strains. Therefore, much of the metabolism represented in iCN718 is maintained in these strains. Three genes were unique to strain AYE within the iCN718 reconstruction: p3ABAYE0029, p2ABYAYE0004, and ABAYE3614. Noting that most of the iCN718 reconstruction was determined to be part of the core metabolic function for all 75 of these strains, we decided to investigate each strain-specific model’s metabolic capabilities. We were additionally interested in analyzing which genes from iCN718 were lost most often (**Figure 5**). The full clustermap of deletions is available in **Supplementary Figure S3**. Genes involved in fatty acid metabolism were by far the most highly represented subsystem exceeding the number of genes in the next highest-represented subsystems, butanoate metabolism and folate biosynthesis, by 47 genes.

Originally, only three of the 74 strain-specific models could simulate growth and the predominantly determining factor of this was the inability to produce lipopolysaccharide (LPS). This result is unsurprising given that LPS is known to vary from strain to strain (Pantophlet et al., 2001). The strains that could still synthesize LPS were A1, AB0057, and AB307-0294, suggesting that these strains may have similar LPS compositions to strain AYE. After recognizing LPS as the main limitation to growth for the majority of the strains, we removed LPS from the biomass function for the remaining strains to investigate other properties. With LPS removed, all but four strains could grow. The four strains unable to grow were, as expected, the four strains with the most deletions from the original AYE model. Interestingly, the one strain that was not isolated from a human, SDF, was instead isolated from lice and required 71 more deletions than the next highest dissimilar strain. This suggests that *Acinetobacter* are indeed highly adaptable to varying environments in their metabolic capabilities and that an expanded pan-genome analysis with a higher number of varied strain environments would yield interesting insights.

We then looked at every strain’s ability to grow in the same minimal media conditions with sole carbon and nitrogen sources on which iCN718 was originally tested. All of the strains that could grow without LPS in the biomass function maintained the carbon and nitrogen catabolic capabilities exhibited by AYE in iCN718. This analysis is limited in that we are dealing with draft strain specific models, which are all derived from the content common to iCN718. To account for additional capabilities of each strain requires more data and deeper study of these strains. However, this approach demonstrates that with one high-quality reconstruction, insight can be gleaned into a large number of strains from their sequences alone.

## Conclusion

*Acinetobacter baumannii* is an urgent clinical threat for which treatment is becoming increasingly difficult. High-quality GEMs of strains of *A. baumannii* can be an important tool to accelerate the advancement of new treatments. We updated and improved a previous reconstruction, AbyMBEL891, to produce a new reconstruction, iCN718. We tested iCN718 on multiple gene essentiality datasets as well as phenotypic microarray data. We demonstrated the utility of iCN718 and GEMs to gain further insight into related strains through their sequences alone. iCN718 is in a standardized and curated format that lends itself to further use by the community studying *Acinetobacter*, as well as in future multi-strain reconstructions of diverse *A. baumannii* strains. We demonstrated that iCN718 represents a significant improvement on AbyMBEL891 and a critical step in the progress toward a truly comprehensive knowledge-base for *A. baumannii*. As the knowledge of this organism continues to grow, iCN718 will provide a platform for the integration of further knowledge and data as well as a tool for future investigations.

## Materials and Methods

### Reconstructing iCN718

We first obtained a draft metabolic reconstruction of *A. baumannii* AYE utilizing the ModelSeed (Henry et al., 2010). AbyMBEL891 was then referenced against this draft reconstruction to compare for the content of each reconstruction. Additional databases (ExPASy, KEGG, MetaNetX, BiGG) were used to refine the reconstruction and obtain a reconstruction utilizing standardized BiGG identifiers (Kanehisa and Goto, 2000; Gasteiger et al., 2003; Ganter et al., 2013; King et al., 2016). The result was a draft reconstruction in BiGG format built upon AbyMBEL891, the draft reconstruction via ModelSeed, and information from the aforementioned databases. To obtain the most accurate final model, this draft reconstruction was then extensively manually curated. This process involved investigating the current literature and rectifying inconsistencies present in the reconstruction. We determined and subsequently filled gaps identified through topological gap analysis and flux-based functional tests. The pathway visualization tool, ESCHER, was instrumental in this gap analysis (King et al., 2015). We also utilized the GrowMatch algorithm to obtain potential reactions to fill identified gaps (Kumar and Maranas, 2009). Additionally, the recently published model of *A. baumannii* ATCC 19606, iLP844, was used as an additional resource for cases of conflicting information amongst the aforementioned sources (Presta et al., 2017). iLP844 was particularly used to check reaction reversibility. The model content was further improved by comparing it to numerous experimental datasets. In particular, iLP844 was used to confirm reaction reversibility. The model content was further improved by comparing it to numerous experimental datasets and making iterative improvements to increase agreement with experimental data. The manual curation was an iterative process and as such was continuously repeated to yield the highest quality reconstruction possible.

### Constraint-Based Modeling

The network reconstruction was converted to a mathematical representation formed from the stoichiometric coefficients of the biochemical reactions. This stoichiometric matrix, **S**, encapsulates in its columns each mass- and charge-balanced reaction of the network, while each row represents a specific metabolite. The model is assumed to be at homeostatic state (Equation 1).

(1) $$S^{*}v\;\text{=}\;\text{0}$$

Thermodynamic constraints for network fluxes are incorporated in the form of bounds that incorporate directionality of reactions. The reconstructed model was analyzed with CoBRApy-0.6.1 (COnstraints-Based Reconstruction and Analysis for Python; Ebrahim et al., 2013) and GLPK 4.32 solver. Flux balance analysis (FBA) is a well-established optimization technique and was used in this study. For a primer on FBA, refer to Orth et al. (2010).

### Gene Essentiality

Gene essentiality predictions were determined by simulating single gene deletions of each applicable gene in the model depending on the dataset in question. Growth of the single gene deletion mutants was predicted using FBA and if, following a gene deletion, there was no growth, this gene was determined to be essential. For all gene-essentiality datasets, the corresponding set of orthologous genes, since no available single gene deletion datasets exist for *A. baumannii* AYE, was obtained via NCBI Bidirectional BLAST (Sayers et al., 2012).

### Growth Conditions

For comparison to experimental data, there were two growth media needed for *in silico* simulations. For any experimental set executed in rich media all exchange reactions were set to -10 mmol.g^-1^.h^-1^ to mimic non-limiting conditions and access to multiple carbon and nitrogen sources. The second media condition utilized was Simmons Minimal Media (Simmons, 1926). The validation of the de Berardinis et al. (2008) gene essentiality dataset and recapitulation of Biolog Phenotypic Array data by Farrugia et al. (2013) each used variations of the Simmons’ Minimal Media (**Supplementary Table S3**). Lower bounds for the exchange reactions of nutrients present in Simmons’ Minimal Media were set to -10 mmol.g^-1^.h^-1^ and the carbon source of interest was also then set to -10 mmol.g^-1^.h^-1^. As described in the experimental protocol by Farrugia et al. (2013) for testing of nitrogen sources, the minimal media was supplemented with xylose.

### Metabolite Connectivity

The stoichiometric matrices of iCN718 and AbyMBEL891 were used to calculate the metabolite connectivities of every species in each network. The metabolite connectivity is a sum of the number of each reaction a metabolite participates in. Metabolite connectivities were then ranked from greatest to least connected to form a discrete distribution (**Supplementary Figure S1**).

### Pan-Genome Analysis

The pan-genome of all 75 completely sequenced strains was constructed by clustering protein sequences based on their sequence homology using the CD-hit package (v4.6). CD-hit clusters protein sequences based on their sequence identity (Li and Godzik, 2006). CD-hit clustering was performed with 0.8 threshold for sequence identity and a word length of 5. A cluster formed by CD-hit is hereon referred to as a gene family. The pan-genome was subdivided into core and accessory genomes. We defined the core genome as gene families that were found in at least 74/75 strains. The subdivided pan-genome was subsequently utilized to identify genes that were part of the core or accessory genome.

## Author Contributions

JM and BP conceived and designed the study. JM, BP, and CN prepared the first draft of the manuscript. CN performed the metabolic network reconstruction and model simulations. All the authors discussed the results and participated in the writing process.

## Conflict of Interest Statement

The authors declare that the research was conducted in the absence of any commercial or financial relationships that could be construed as a potential conflict of interest. The handling Editor declared a past co-authorship with the authors BP and JM.

## Supplementary Material

FIGURE S1 Calculated metabolite connectivity for **(A)** iCN718 and **(B)** AbyMBEL891. The dashed orange line serves as a reference and points above the line indicate strong connectivity.

FIGURE S2 COG classifications for the pan-genome content of the 75 strains. Note that this only represents those genes that could be COG classified which is only half of the entire set designated in the pan-genome.

FIGURE S3 Full clustermap of presence or absence for all genes in strain specific models of each of the 75 strains.

TABLE S1 Comparison between carbon source Biolog Phenotypic Array data and *in silico* outcomes.

TABLE S2 Comparison between nitrogen source Biolog Phenotypic Array data and *in silico* outcomes.

TABLE S3 Simmons minimal media composition *in silico*.

TABLE S4 Full double gene deletion results.

TABLE S5 Synthetic lethal gene pairs.

TABLE S6 Genome IDs and strain names used for pan-genome analysis.

TABLE S7 Genes knocked out for each strain to create draft strain specific models.

DATA SHEET S1 iCN718.xls: Reconstruction in spreadsheet format.

DATA SHEET S2 iCN718.json: Reconstruction in JSON format.

DATA SHEET S3 iCN718.sbml: Reconstruction in SBML format.
