# Supplementary material for: iCN718, an Updated and Improved Genome-Scale Metabolic Network Reconstruction of Acinetobacter baumannii AYE
Source: Front Genet. 2018 Apr 10;9:121. doi: 10.3389/fgene.2018.00121 (PMC5902709; doi:10.3389/fgene.2018.00121)
Supplement: FIGURE S1 — Calculated metabolite connectivity for (A) iCN718 and (B) AbyMBEL891. The dashed orange line serves as a reference and points above the line indicate strong connectivity. [file Image_1.PDF]

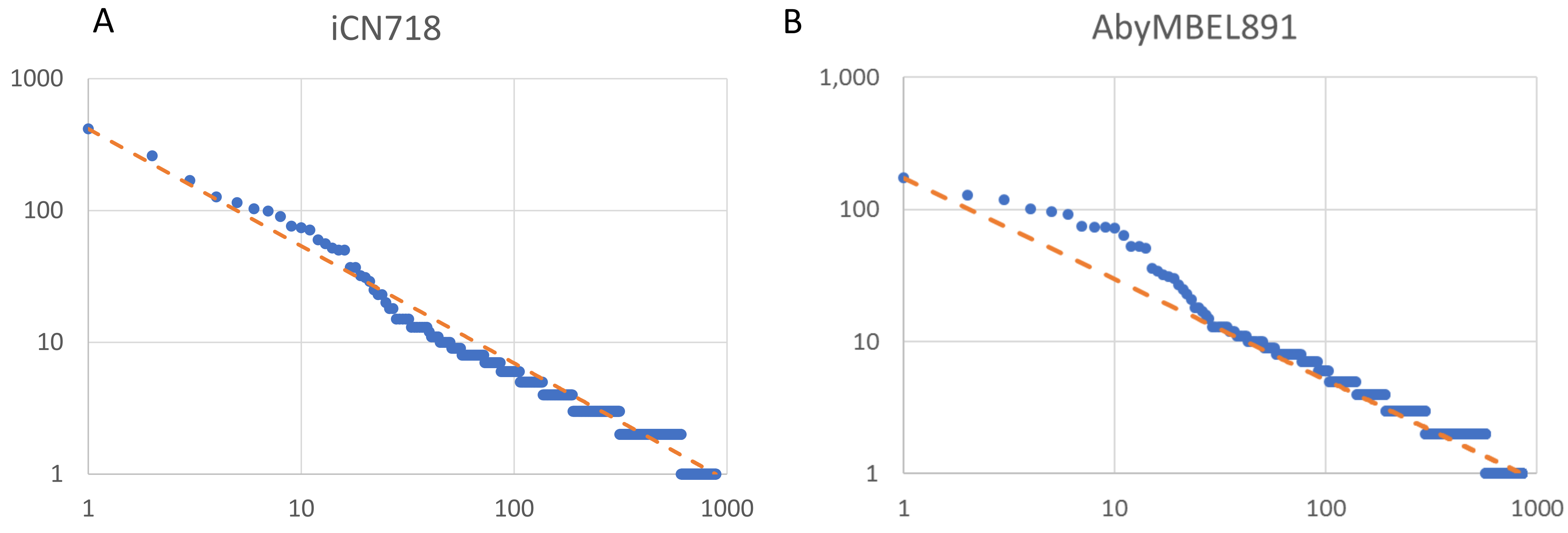

Supplementary Figure 1: Calculated metabolite connectivity for (A) iCN718 and (B) AbyMBEL891. The dashed orange line serves as a reference and points above the line indicate strong connectivity.
