# Supplementary material for: iCN718, an Updated and Improved Genome-Scale Metabolic Network Reconstruction of Acinetobacter baumannii AYE
Source: Front Genet. 2018 Apr 10;9:121. doi: 10.3389/fgene.2018.00121 (PMC5902709; doi:10.3389/fgene.2018.00121)
Supplement: FIGURE S2 — COG classifications for the pan-genome content of the 75 strains. Note that this only represents those genes that could be COG classified which is only half of the entire set designated in the pan-genome. [file Image_2.PDF]

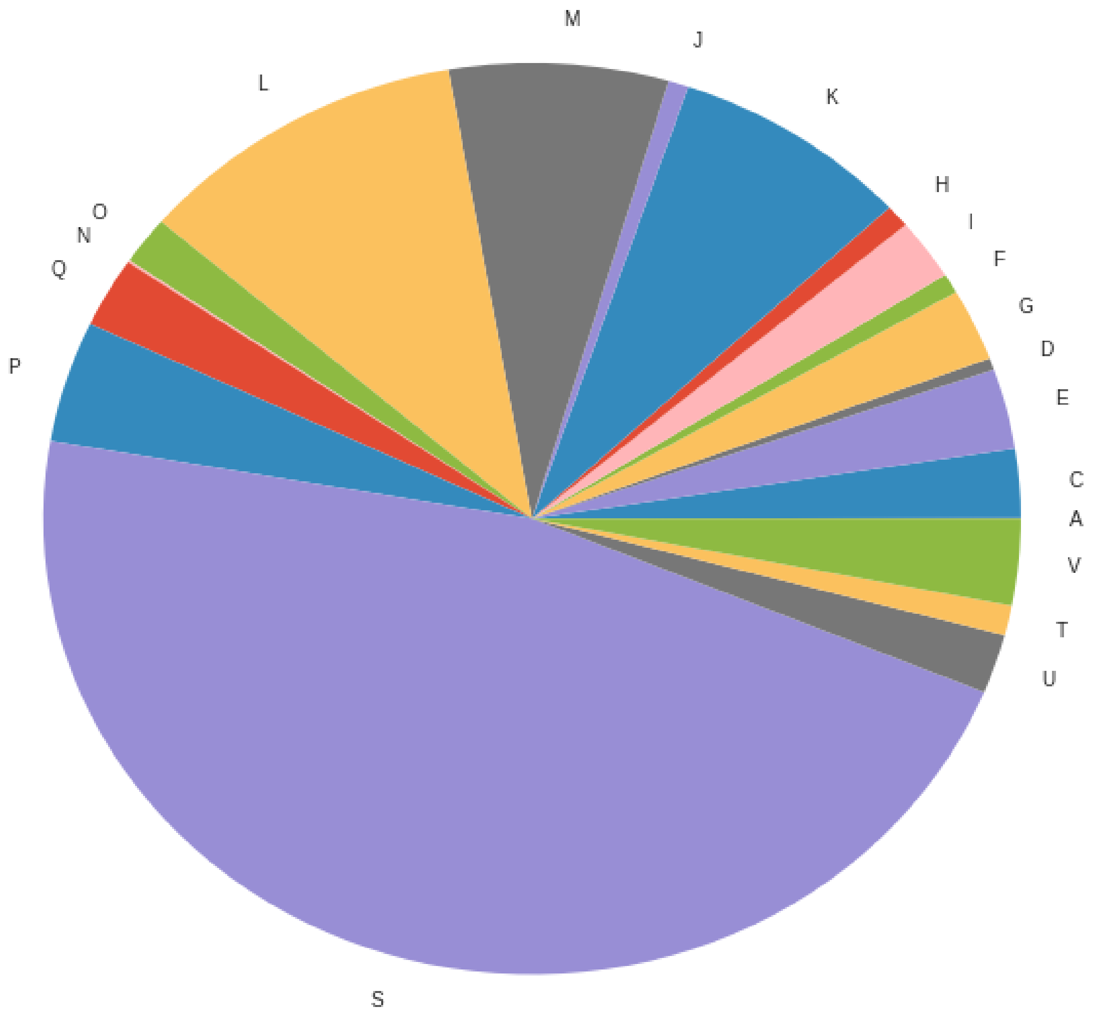

Supplementary Figure 2: COG classifications for the pan-genome content of the 75 strains. Note that this only represents those genes that could be COG classified which is only half of the entire set designated in the pan-genome.
