# Supplementary material for: iCN718, an Updated and Improved Genome-Scale Metabolic Network Reconstruction of Acinetobacter baumannii AYE
Source: Front Genet. 2018 Apr 10;9:121. doi: 10.3389/fgene.2018.00121 (PMC5902709; doi:10.3389/fgene.2018.00121)
Supplement: FIGURE S3 — Full clustermap of presence or absence for all genes in strain specific models of each of the 75 strains. [file Image_3.PDF]

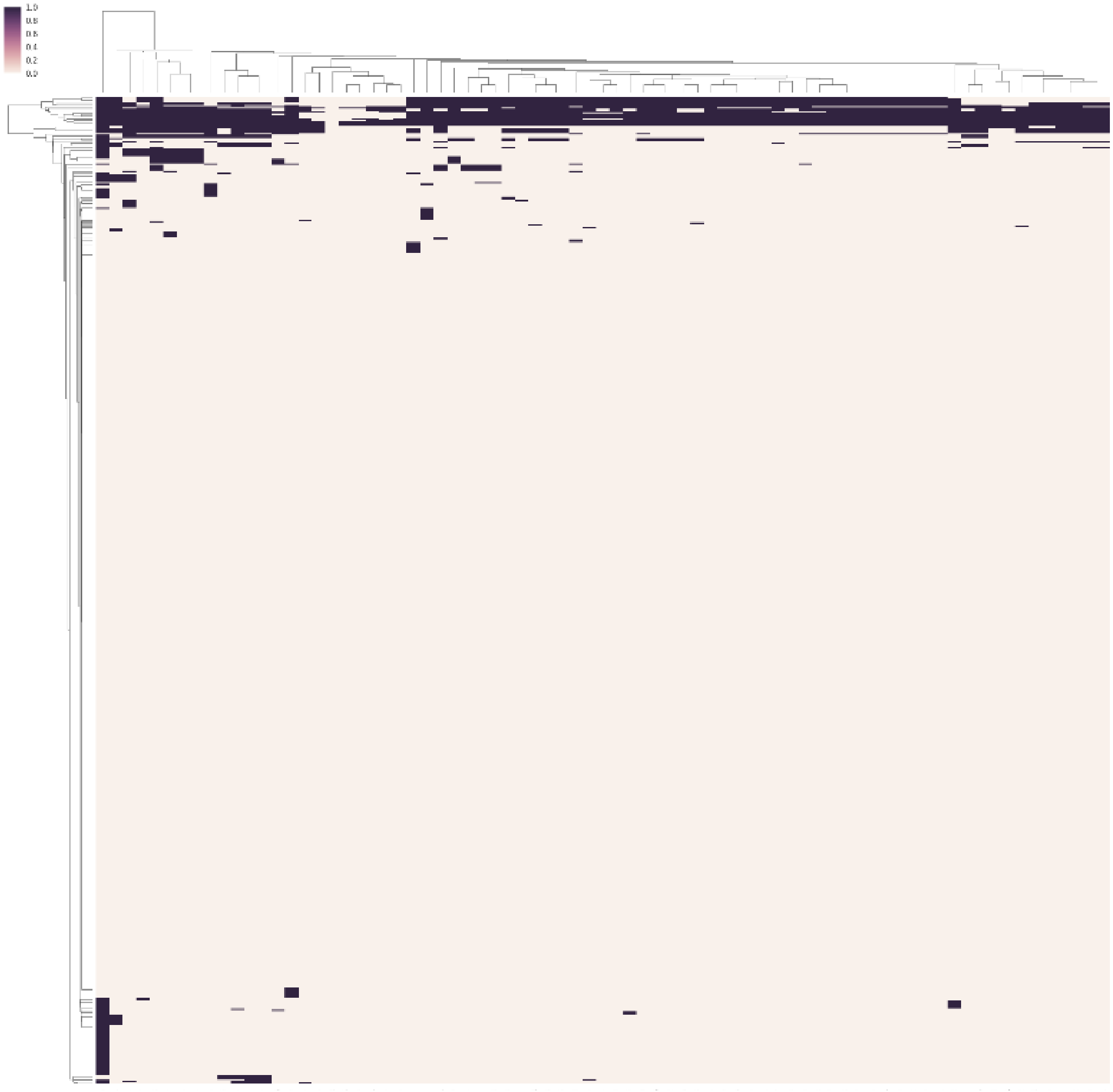

Supplementary Figure 3*:* Full clustermap of presence or absence for all genes in strain specific models of each of the 75 strains.
